# Design of a New Method for Detection of Occupancy in the Smart Home Using an FBG Sensor

**DOI:** 10.3390/s20020398

**Published:** 2020-01-10

**Authors:** Jan Vanus, Jan Nedoma, Marcel Fajkus, Radek Martinek

**Affiliations:** 1 Department of Cybernetics and Biomedical Engineering, Faculty of Electrical Engineering and Computer Science, VSB–Technical University of Ostrava, 708 33 Ostrava, Czech Republic; radek.martinek@vsb.cz; 2 Department of Telecommunications, Faculty of Electrical Engineering and Computer Science, VSB–Technical University of Ostrava, 708 33 Ostrava, Czech Republic; jan.nedoma@vsb.cz (J.N.); marcel.fajkus@vsb.cz (M.F.)

**Keywords:** smart home (SH), prediction, artificial neural network (ANN), fiber bragg grating (FBG), occupancy, number of person recognition, scaled conjugate gradient (SCG)

## Abstract

This article introduces a new way of using a fibre Bragg grating (FBG) sensor for detecting the presence and number of occupants in the monitored space in a smart home (SH). CO_2_ sensors are used to determine the CO_2_ concentration of the monitored rooms in an SH. CO_2_ sensors can also be used for occupancy recognition of the monitored spaces in SH. To determine the presence of occupants in the monitored rooms of the SH, the newly devised method of CO_2_ prediction, by means of an artificial neural network (ANN) with a scaled conjugate gradient (SCG) algorithm using measurements of typical operational technical quantities (indoor temperature, relative humidity indoor and CO_2_ concentration in the SH) is used. The goal of the experiments is to verify the possibility of using the FBG sensor in order to unambiguously detect the number of occupants in the selected room (R104) and, at the same time, to harness the newly proposed method of CO_2_ prediction with ANN SCG for recognition of the SH occupancy status and the SH spatial location (rooms R104, R203, and R204) of an occupant. The designed experiments will verify the possibility of using a minimum number of sensors for measuring the non-electric quantities of indoor temperature and indoor relative humidity and the possibility of monitoring the presence of occupants in the SH using CO_2_ prediction by means of the ANN SCG method with ANN learning for the data obtained from only one room (R203). The prediction accuracy exceeded 90% in certain experiments. The uniqueness and innovativeness of the described solution lie in the integrated multidisciplinary application of technological procedures (the BACnet technology control SH, FBG sensors) and mathematical methods (ANN prediction with SCG algorithm, the adaptive filtration with an LMS algorithm) employed for the recognition of number persons and occupancy recognition of selected monitored rooms of SH.

## 1. Introduction

Recognizing the occupancy, number of individuals, location-and-movement recognition and activity recognition of an individual in an indoor space is one the key functionalities of a smart home (SH), as a prerequisite to providing services to support independent living of elderly SH occupants and has a great influence on internal loads and HVAC (heating, ventilation and air conditioning) requirement, thus increasing the energy consumption optimization. Azghandi et al. focused on the particular case of an SH with multiple occupants, they developed a location-and-movement recognition method using many inexpensive passive infrared (PIR) motion sensors and a small number of more costly radio frequency identification (RFID) readers [1]. Benmansour et al. provided an overview of existing approaches and current practices for activity recognition in multi-occupant SHs [2]. Braun et al. reported the investigation of two categories of occupancy sensors with the requirements of supporting wireless communication and a focus on the low cost of the systems (capacitive proximity sensors and accelerometers that are placed below the furniture) with a classification accuracy between 79% and 96% [3]. Chan et al. proposed the methodology and design of a voice-controlled environment, with an emphasis on speech recognition and voice control, based on Amazon Alexa and Raspberry Pi in an SH [4]. Chen et al. proposed an activity recognition system guided by an unobtrusive sensor (ARGUS) with a facing direction detection accuracy, resulting from manually defined features, that reached 85.3%, 90.6%, and 85.2% [5]. Khan et al. developed a low-cost heterogeneous radar-based activity monitoring (RAM) system for recognizing fine-grained activities in an SH with detecting accuracy of 92.84% [6]. Lee et al. investigated the use of cameras and a distributed processing method for the automated control of lights in an SH, which provided occupancy reasoning and human activity analysis [7]. Mokhtari et al. proposed a new human identification sensor, which can efficiently differentiate multiple residents in a home environment to detect their height as a unique bio-feature with three sensing/communication modules: pyroelectric infrared (PIR) occupancy, ultrasound array, and Bluetooth low-energy (BLE) communication modules [8]. A new recognition algorithm for household appliances, based on a Bayes classification model, is presented by Yan et al., in which sequential appliance power consumption data from intelligent power sockets is used and for the generalization and extraction of the characteristics of occupant behavior and power consumption of typical household appliances [9]. Yang et al. proposed a novel indoor tracking technique for SHs with multiple residents by relying only on non-wearable, environmentally deployed sensors such as passive infrared motion sensors [10]. Feng et al. presented a novel real-time, device-free, and privacy-preserving WiFi-enabled Internet of Things (IoT) platform for SH-occupancy sensing, which can promote a myriad of emerging applications with an accuracy of 96.8% and 90.6% in terms of occupancy detection and recognition, respectively [11]. Traditionally, in building energy modeling (BEM) programs, occupant behavior (OB) inputs are deterministic and less indicative of real-world scenarios, contributing to discrepancies between simulated and actual energy use in buildings. Yin et al. (2016) presented a new OB modeling tool, with an occupant behavior functional mock-up unit (obFMU) that enables co-simulation with BEM programs implementing a functional mock-up interface (FMI) [12]. Occupants are involved in a variety of activities in buildings, which drive them to move among rooms, enter or leave a building. Hong et al. (2016) defined SH occupancy using four parameters and showed how they varied with time. The four occupancy parameters were as follows: (1) the number of occupants in a building, (2) occupancy status of space, (3) the number of occupants in a space, and (4) the location of an occupant [13].

In order to detect the occupancy of the SH by indirect methods (without using cameras), common operational and technical sensors are used in this article to measure the indoor temperature, the indoor relative humidity and the CO_2_ indoor concentration within the BACnet technology for HVAC control. A fibre Bragg grating (FBG) sensor will be used to detect the number of occupants in the monitored space of room R104 (ground floor). The method devised for the prediction of the CO_2_ waveform using artificial neural network (ANN) scaled conjugate gradient (SCG) will be verified during the experiments conducted to detect the occupancy of rooms R104, R203 and R204. The input quantities measured to ANN SCG were obtained from the indoor temperature and relative indoor humidity sensors. One of the objectives of the article is to verify the possibility of minimizing investment costs by using cheaper temperature and relative humidity sensors instead of a more expensive CO_2_ sensor to detect the occupancy of monitored SH spaces. The other objectives of this article are the following:

1. Experimental verification of FBG sensor use for the recognition of the number occupants in SH room R104.
2. Experimental verification of the CO_2_ concentration measurement in an SH by means of common operational sensors for the occupancy status of the SH space.
3. Experimental verification of the method with ANN SCG that was devised for CO_2_ concentration prediction (more one-day measurements in the period from 25 June 2018, to 28 June 2018) to locate an occupant (in rooms R104, R203, and R204) in an SH with the highest possible accuracy.
4. Experimental verification of the possibility of ANN learning for one room only (R203) in order to predict CO_2_ concentrations in other rooms (R104, R204).

**Figure 1:**
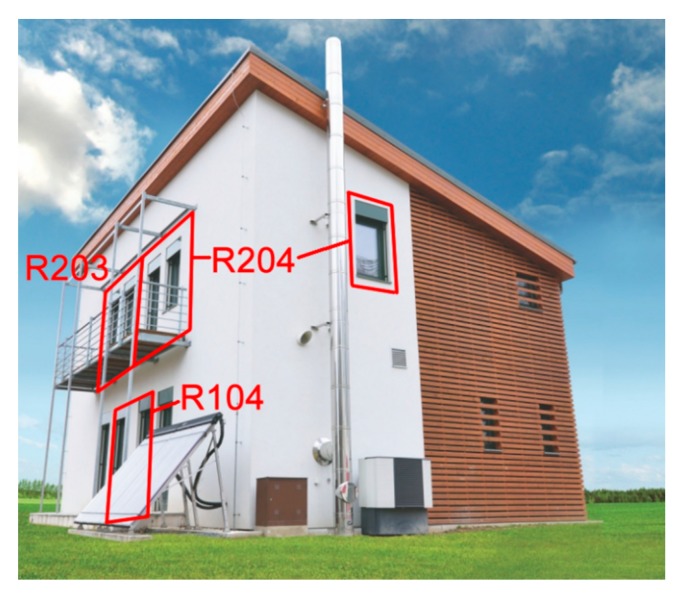
The wooden building of the passive standard located in the Faculty of Civil Engineering, VSB—TU Ostrava with selected smart home (SH) rooms R204, R203, and R104 [15].

The experimental measurements of objective parameters of the internal environment and thermal comfort evaluation were conducted in selected SH rooms R204, R203, and R104 in a wooden building of the passive standard located in the Faculty of Civil Engineering, VSB—TU Ostrava. (Figure 1) [14].

## 2. Materials and Methods

### 2.1. Fiber Bragg Grating (FBG) Sensor Using for Recognition of Number Occupants in Smart Home (SH) Room R104

Bragg gratings (FBG) are special structures created by an ultraviolet (UV) laser inside the core of a photosensitive optical fibre. This structure consists of a periodic structure of changes in the refractive index, where the layers of the refractive index of the core $n_{1}$ alternate with the layers of the increased refractive index $n_{3}$ (1):

(1) $$n_{3}=n_{1}+\delta n$$

where δn is the refractive index induced by UV radiation [16].

**Figure 2:**
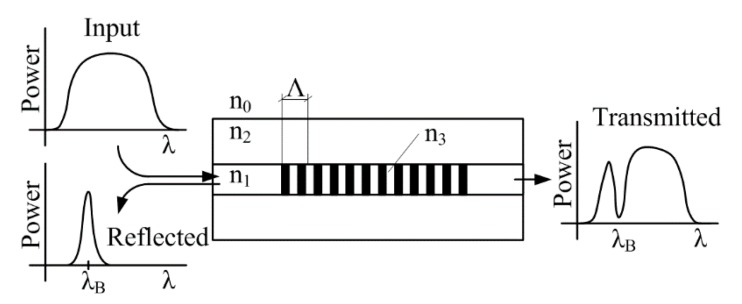
The Bragg grating principle.

When a broad-spectrum light is introduced into the optical fibre, the Bragg grating reflects a narrow spectral portion and all the other wavelengths pass through the structure without damping (Figure 2).

The central wavelength of the reflected spectral portion is called the Bragg wavelength $\lambda_{B}$ and is defined by the optical and geometric properties of the structure according to (2):

(2) $$\lambda_{B}=2n_{eff}\Lambda$$

where $n_{eff}$ is the effective refractive index of the periodic structure and Λ is the distance between the periodic changes in the refractive index. The external effects of the temperature and the deformation influence the optical and geometric properties and thus, the spectral position of the Bragg wavelength. Thanks to this feature, Bragg gratings are used in sensory applications. The dependence of the Bragg wavelength on the deformation and the temperature is expressed by (3):

(3) $$\frac{\Delta \lambda_{B}}{\lambda_{B}}=k\varepsilon +(\alpha_{\Lambda }+\alpha_{n})\Delta T$$

where k is the deformation coefficient, ε is the optical fibre deformation caused by measurement, $\alpha_{\Lambda }$ is the coefficient of thermal expansion, $\alpha_{n}$ is the thermo-optic coefficient and ΔT is the change in the operating temperature [17].

Bragg gratings in a standard optical fibre with a central wavelength of 1550 nm show a deformation sensitivity of 1.1 pm/µstrain and a temperature sensitivity of 10.3 pm/°C. By using a suitable encapsulation, it is possible to implement a sensor of almost any physical quantity. FBG sensors are used in automobile [18] and railway transport [19], the construction industry [20], power engineering, biomedical [21] or perimetric applications [22], etc.

Bragg gratings are single-point sensors. By using a wavelength or time multiplex, it is possible to connect tens or hundreds of these sensors in a single optical fibre in order to achieve a quasi-distributed sensory system [23].

#### 2.1.1. Fiberglass Bragg Sensors

One of the most widespread applications of Bragg gratings includes deformation and compression measurements. Depending on the type of application, Bragg gratings can be encapsulated in many ways. The principle of the encapsulation is to protect the fragile glass fibre, to enhance the sensitivity to the desired quantity and to suppress the surrounding interference. Bragg gratings can be encapsulated in polymers [24] of fibreglass or composite materials [25,26], and special steel jigs [27], which are mechanically attached to the structure that is to be measured, etc. Based on the advantages of using Bragg gratings—such as their reliable and very accurate measurement—these types of sensors were used for the reference measurement of the occupancy recognition of room R104 in the SH.

Because the grating sensors were installed on a wooden staircase, the encapsulation of Bragg gratings in fibreglass strips was used. This method enables the implementation of a very thin sensor that transmits deformations from the step (passage of persons) to the optical fibre itself.

**Figure 3:**
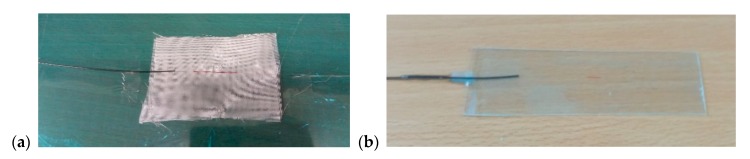
Implementation of the sensor by encapsulating the Bragg grating in fibreglass (**a**); the resulting fibre Bragg grating (FBG) fibreglass sensor (**b**).

Two Bragg gratings in a single-mode optical fibre with primary acrylate protection were used for implementing the sensors. Bragg gratings A and B had the following parameters: The Bragg wavelengths were 1547.510 nm and 1552.369 nm, respectively; the reflection spectrum width was 256 pm and 227 pm, respectively; the reflectivity was 91.2% and 91.3%, respectively. Each Bragg grating was placed between the glass fabrics (2 layers below and 2 layers above the optical fibre). The glass fabric was then coated with a polymer resin. The actual curing caused a Bragg wavelength shift of 23 µm for Sensor A and of 19 µm for Sensor B to lower wavelengths (Figure 3). The Bragg grating is located in the middle of the fibreglass strip, marked in red.

#### 2.1.2. Implementation of FBG Sensors

**Figure 4:**
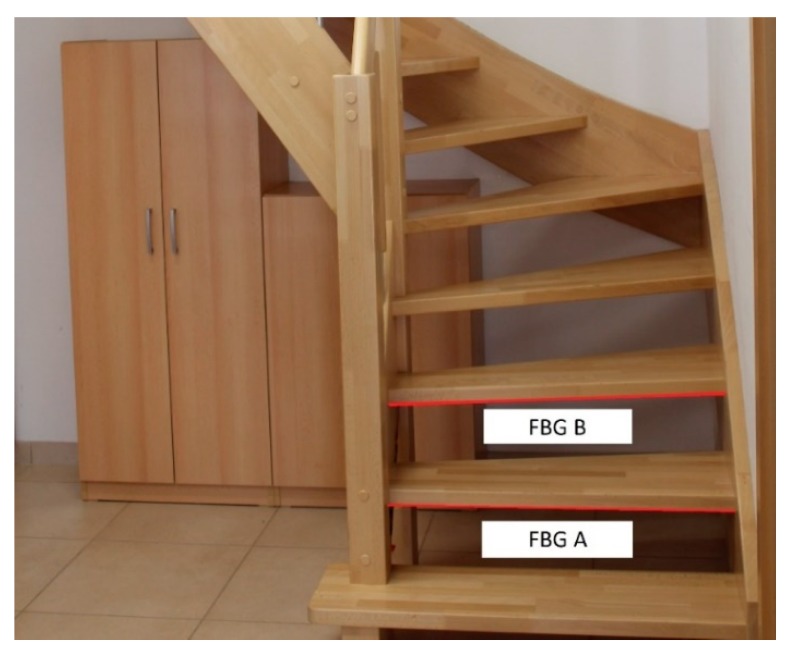
Placement of FBG sensors on the staircase in the smart home (SH), room R104.

FBG sensors were implemented on the staircase leading from the ground floor to the first floor (Figure 4). The sensors were glued with cyanoacrylate adhesive to the bottom of the second step (FBG A) and the third step (FBG B).

### 2.2. Use of a CO_2_ Sensor Network for Monitoring SH Space Occupancy

**Figure 5:**
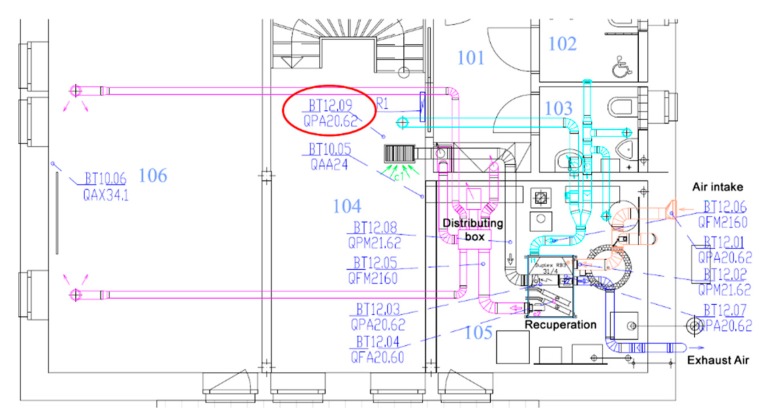
The ground plan of the ground floor of the SH with the location of the sensors for CO_2_ measurement.

**Figure 6:**
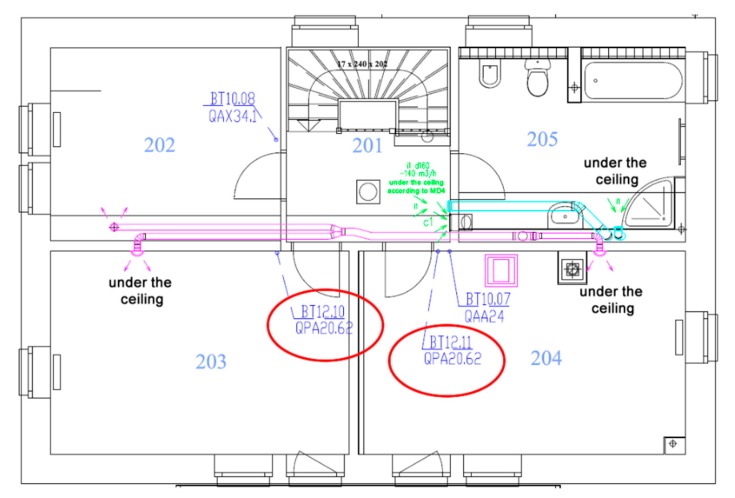
The ground plan of the first floor of the SH with the location of the sensors for CO_2_ measurement.

**Figure 7:**
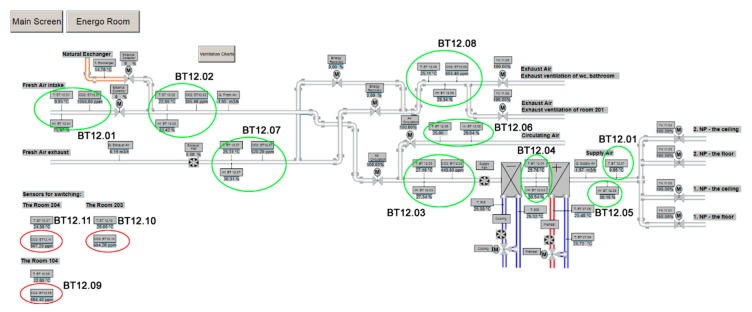
The ventilation distribution technology with the location of the individual sensors for CO_2_ measurement in an SH.

**Figure 8:**
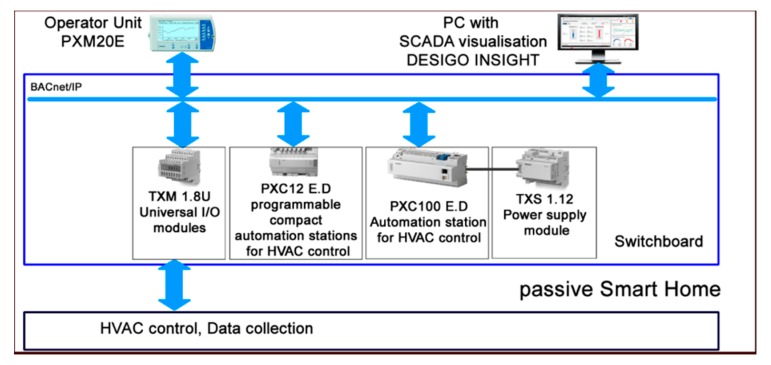
Block diagram of the BACnet technology used in an SH for HVAC control.

Common CO_2_ sensors can be used to detect the occupancy of individual SH spaces. In room R104, the BT 12.09 sensor (Figure 5), in room R203 the BT 12.10 sensor and in room R204 the BT 12.10 sensor (Figure 6) were used for measuring the CO_2_ concentration in the framework of forced Air Condition (AC) control (Figure 7). The BACnet technology is used in the SH to control HVAC (Figure 8). The presence of occupants in the SH can be detected by measuring the CO_2_ concentration. Figure 5 shows the ground plan of the ground floor of the SH with the location of the individual sensors for CO_2_ measurement.

The individual rooms on the ground floor of the SH are marked as follows (Figure 5):

- R101—door space, entrance hall,
- R102—toilet 1,
- R103—toilet 2,
- R104—entrance room; FBG sensor is placed on the staircase,
- R105—utility room, there are heating sources,
- R106—classroom.

Figure 6 shows the ground plan of the first floor of the SH with the location of the individual sensors for CO_2_ measurement.

The individual rooms on the SH first floor are marked as follows (Figure 6):

- R201—staircase,
- R202—control room,
- R203—classroom (office),
- R204—classroom (office),
- R205—toilet and bathroom.

The list (legend) of the individual sensors used (Figure 5, Figure 6 and Figure 7):

- BT 12.01—Measurement at the fresh outdoor air inlet into QPA 2062 SH.
- BT 12.02—Measurement at the recirculation air inlet from SH spaces into QPM 2162 heat recovery unit.
- BT 12.03—Measurement at the recirculation air inlet from SH spaces into QPA 2062 heat recovery unit.
- BT 12.04—Measurement in QFA 2060 heat recovery unit.
- BT 12.05—Measurement at the recirculation and fresh air outlet from the heat recovery unit into QFM 2160 SH.
- BT 12.06—Measurement at the exhaust air inlet into QFM 2160 recuperation unit.
- BT 12.07—Measurement at the exhaust air outlet from QPA 2062 recuperation unit.
- BT 12.08—Measurement at the recirculation air inlet from SH spaces into QPM 2162 heat recovery unit.
- BT 12.09—sensor located in room R104, QPA 2062.
- BT 12.10—sensor located in room R203, QPA 2062.
- BT 12.11—sensor located in room R204, QPA 2062.

The technical specification of the individual sensors used:

- QPA 20.62 room sensor for measuring the air quality—CO_2_, relative humidity and temperature—with a measurement accuracy: (50 ppm + 2% of the value measured, long-term drift: 5% of the measuring range/5 years (typically). The CO_2_ sensor principle is based on non-dispersive infrared absorption (NDIR) measurement.
- QPM 21.62 channel sensors for air quality—CO_2_, relative humidity, temperature. Measurement accuracy: (50 ppm + 2% of the value measured), long-term drift: 5% of the measuring range/5 years (typically). The CO_2_ sensor is based on non-dispersive infrared absorption (NDIR) measurement.
- QFA 20.60 room sensor for temperature and relative humidity. Measurement accuracy ± 3% rH_in_ within the comfort range. Application range −15 … +50 °C/0 … 95% rH_in_ (no condensation).
- QFM 21.60 channel sensor for relative humidity and temperature. Measurement accuracy ± 3% rH_in_ within the comfortable range. Application range −15 … +60 °C/0 … 95% rH_in_ (no condensation).

Figure 7 shows the ventilation distribution technology with the location of the individual CO_2_ sensors.

A block diagram containing a description of the individual components and function blocks within the BACnet technology in the SH for HVAC control is shown in Figure 8.

### 2.3. The Design of the New Method for CO_2_ Prediction

**Figure 9:**
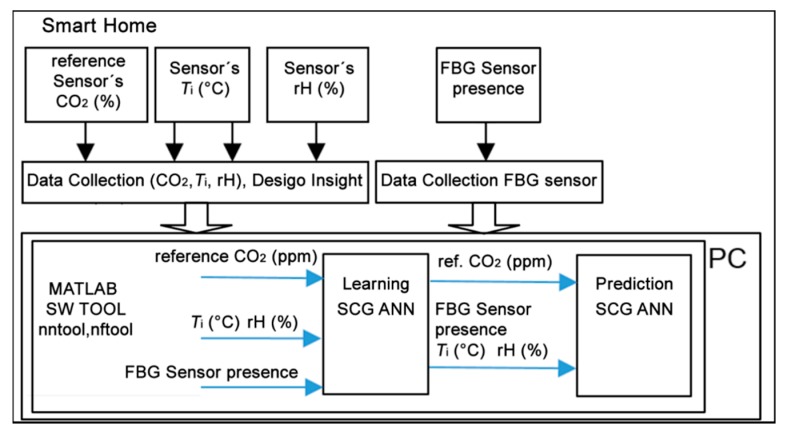
Block diagram describing processing of the data measured by means of a scaled conjugate gradient artificial neural network (ANN SCG) within the method devised for CO_2_ prediction.

The newly devised method for CO_2_ prediction from the temperature indoor and relative humidity indoor values measured by means of ANN SCG (multiple one-day measurements) was used for the location of an occupant (in rooms R104, R203 a R204) in SH with the highest possible accuracy. Block diagram of processing the quantities measured in SH for multiple one-day measurements in the period from 25 June 2018, to 28 June 2018, using the method devised for CO_2_ prediction by means of ANN SCG is shown in Figure 9.

**Figure 10:**
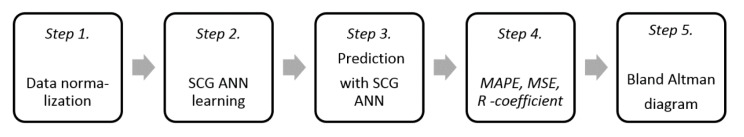
Block scheme summarizing the experiment steps.

The measured values that were used in these experiments are the indoor CO_2_ concentration, indoor relative humidity and indoor temperature. The data were pre-processed to improve the efficiency of neural network training. That means that data were normalized so that all the values are between 0 and 1 (Figure 10, Step 1).

In Matlab, the function nftool (neural fitting) was used with neurons varying from 10 to 100 and the three methods previously mentioned. The data samples were divided into 3 sets: training (used to teach the network), validation and testing (provides an independent measure of the network training) (Figure 10, Step 2). After training the networks with data measured on 25 July for room 203, the 90 networks were used to predict data for the rest of the dates from the same room, as well as the two others. Since the learning date was different from the prediction dates, this was called “cross-validation”. In this step, the function used in Matlab was ‘nntool’ (Figure 10, Step 3). Once the results were ready, the next step was to calculate some parameters that would allow us to quantify the precision of the results, and, therefore, compare the prediction quality between the different ANN SCG (Figure 10, Step 4).

The three parameters we relied on for our experiments are as follows.

R (correlation coefficient) is a statistical measure that calculates the strength of the relationship between the relative movements of two variables and is calculated with the formula (4). The values range between −1 and 1. A value of exactly 1.0 means there is a perfect positive relationship between the two variables. For a positive increase in one variable, there is also a positive increase in the second variable. A value of −1.0 means there is a perfect negative relationship between the two variables. This shows that the variables move in opposite directions—for a positive increase in one variable, there is a decrease in the second variable. If the correlation is 0, there is no relationship between the two variables [28].

(4) $$R=\frac{\sum \left(x-\bar{x}\right)\left(y-\bar{y}\right)}{\sqrt{\sum {\left(x-\bar{x}\right)}^{2}\sum {\left(y-\bar{y}\right)}^{2}}}$$

The MSE (mean squared error) parameter describes how close a regression line is to a set of points and is calculated with formula (5). It does this by taking the distances from the points to the regression line (these distances are the “errors”) and squaring them. The squaring is necessary to remove any negative signs. It also gives more weight to larger differences. It is called the mean squared error as you are finding the average of a set of errors [29]:

(5) $$MSE=\;\frac{1}{n}\overset{n}{\underset{i=1}{\sum }}\left(y_{i}-y_{i}^{*}\right)$$

MAPE (average absolute percentage error) is a statistical measurement parameter of how accurate a forecast system is. It measures this accuracy as a percentage, and it can be calculated as the average absolute percent error for each time period minus actual values divided by actual values which are given by (6) [30]:

(6) $$MAPE=\;\frac{1}{n}\overset{n}{\underset{i=1}{\sum }}\frac{\left|y_{i}-y_{i}^{*}\right|}{y_{i}^{*}}$$

$y_{i}$: reference value,

$y_{i}^{*}$: predicted value,

n: total number of values,

$\bar{x},\bar{y}$: mean of x, y.

After calculating the MAPE, MSE, and R correlation parameters, we plot two figures. The first one has a reference and predicted CO_2_ over time (Figures 18, 20 and 22) and the second one is a Bland–Altmann plot (Figures 19, 21 and 23). The Bland–Altman technique allows us to make a comparison between two measurement methods of the same sample. It was established by J. Martin Bland and Douglas G. Altman. Essentially, it quantifies the difference between measurements using a graphical method. It consists of a scatterplot with the average and the difference represented on the *X*-axis and the *Y*-axis, respectively. The plot also has horizontal lines drawn at the mean difference and at the limits of agreement, which are defined as the mean difference plus and minus 1.96 times the standard deviation of the differences (Figure 10, Step 5). The final step is to compare the results and the plots, so as to decide which conditions and which methods are optimal for future predictions.

The procedure used to carry out the learning process in the neural networks is called optimization algorithms. In the following experiments, a ANN SCG type of mathematical algorithm was used to train the ANNs.

**Scaled Conjugate Gradient Algorithm:**

SCG is a supervised learning algorithm for feedforward neural networks and is a member of the class of conjugate gradient methods (CGMs). Let $p=\exp\left(\frac{-\Delta E}{T}\right)$ be a vector, N the sum of the number of weights and of the number of biases of the network, and *E* the error function we want to minimize. SCG differs from other conjugate gradient methods in two ways [31]:

Each iteration k of a CGM computes $\omega_{i}$, where $R^{N}$ is a new conjugate direction, and $\omega_{k+1}=\omega_{k}+\alpha_{k}.p_{k}$ is the size of the step in this direction. In fact, $p_{k}$ is a function of $\alpha_{k}$, the Hessian matrix of the error function, namely the matrix of the second derivatives. In contrast to other CGMs that avoid the complex computation of the Hessian and approximate $\alpha_{k}$ with a time-consuming line search procedure, SCG makes the following simple approximation of the term $E^{\prime \prime }\left(\omega_{k}\right)$, a key component of the computation of $\alpha_{k}$: $s_{k}$ [32] as the Hessian is not always positive, which prevents the algorithm from achieving good performance; SCG uses a scalar $\alpha_{k}$ which is supposed to regulate the indefiniteness of the Hessian. This resembles the Levenberg–Marquardt method, and is performed by using the following equation (7) [33]:

(7) $$s_{k}=E^{\prime \prime }\left(\omega_{k}\right).p_{k}\approx \frac{E^{\prime }\left(\omega_{k}+\alpha_{k}.p_{k}\right)-E^{\prime }\left(\omega_{k}\right)}{\alpha_{k}},\;0<\alpha_{k}\ll 1$$

and adjusting $\lambda_{k}$ at each iteration.

The final step is to compare the results and the plots, so as to decide which conditions and which methods are optimal for future predictions [34].

### 2.4. The Signed–Regressor LMS Adaptive Filter

The signed–regressor LMS adaptive filter was used to filter the predicted course in order to determine the occupancy of the monitored areas more precisely (Figures 19, 21, 23, 26, 28 and 30).

#### 2.4.1. The Conventional LMS Algorithm

The LMS algorithm is a linear adaptive filtering algorithm, which consists of two basic processes:

(a) a filtering process, which involves computing the output *y*(*n*) of the linear filter in response to an input signal *x*(*n*) (8), generating an estimation error *e*(*n*) by comparing this output *y*(*n*) with the desired response *d*(*n*) (9),
(b) an adaptive process (10), which involves the automatic adjustment of the parameters ***w***(*n*+1) of the filter in accordance with the estimation error *e*(*n*).
  (8) $$y(n)=\sum_{i=0}^{M-1}w_{i}\left(n\right)x\left(n-i\right)$$
  (9) e(n)=d(n)−y(n)
  (10) w(n+1)=w(n)+2μe(n)x(n)
  where **w**(*n*) is *M* tap—weight vector, **w**(*n* + 1) is *M* tap—weight vector update [35,36].

#### 2.4.2. The Signed–Regressor LMS Algorithm

The signed–regressor algorithm is obtained from the conventional recursion (10) by replacing the tap-input vector **x**(*n*) with the vector sign(**x**(*n*)), where the sign function is applied to the vector **x**(*n*) on an element-by-element basis. The signed–regressor recursion is then [35]:

**w**(*n*+1) = **w**(*n*) + 2*μe*(*n*)sign(**x**(*n*)) (11)

## 3. Experiments and Results

### 3.1. Using Fiber Bragg Grating Sensor for Recognition of Number of Occupants in SH Room R104

**Figure 11:**
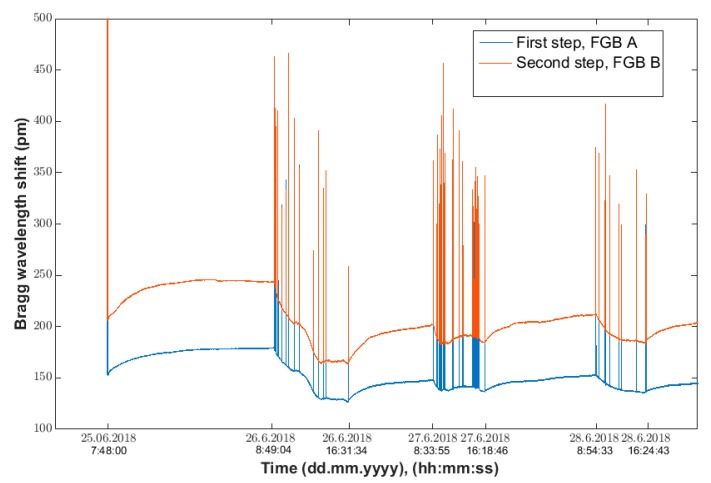
The waveform of signals from FBG A and FBG B sensors during the 24-h measurement of recognition of the number occupants in room R104 in the period from 25 June 2018 (7:48:00), to 28 June 2018 (23:59:00).

The results of the experiments to detect the number of occupants in the monitored space of room R104 are described below. On 25 June 2018, we installed the individual sensors (FBG A and FBG B) on the staircase in room R104 (Figure 4). The actual measurement to unambiguously detect the number of occupants in the monitored space took place from 25 to 28 June 2018. The record of the continuous measurement conducted in the period from 25 to 28 June 2018, is shown in Figure 11. These are signals from the Bragg sensors where the individual peaks represent persons treading on a particular step. Blue shows the waveform of the FBG A sensor on the second step and red shows the signal from FBG B sensor on the third step.

**Figure 12:**
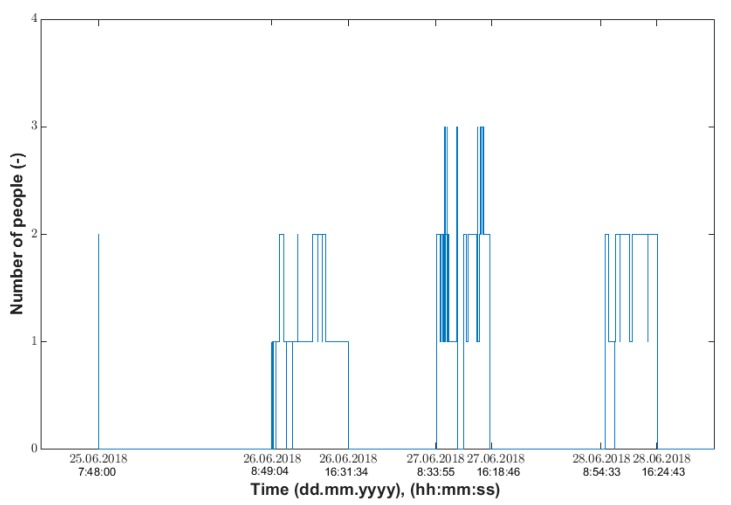
First floor of the recognition of the number occupants obtained from the measurement conducted in the period from 25 June 2018 (7:48:00), to 28 June 2018 (23:59:00).

To detect the number of occupants on the first floor, an algorithm was implemented in the MATLAB (Matrix Laboratory) computational software environment. The algorithm is based on the detection of peaks (treading on the step) from both FBG sensors. The time-corresponding peaks were compared over time, while the direction of the passage (up or down) was detected based on earlier treading on the first or second step. The number of occupants on the first floor was detected using a counter. The occupancy waveform of the first floor is shown in Figure 12.

The number of occupants can be detected from the following Table 1:

**Discussion—Experiment 3.1:**

On the basis of measuring the movement of occupants on the staircase in room R104 using FBG sensors in the period from 26 to 29 June 2019, it is possible to clearly identify the number of occupants (Figure 12) in the monitored SH space (Figure 4)—specifically in room R104 (Figure 5)—with an accuracy of 1 s. Within the initial testing of the experiment conducted, two persons walking up the stairs to the first floor of the SH were detected on 25 June 2018, when installing the FBG sensors in the SH (room R104) (Figure 12), (Table 1). On 26 June 2018, movement of one and two persons on the staircase from the ground floor to the first floor was detected during the day (Figure 12), (Table 1). On 27 June 2018, and 29 June 2018, movement of one, two and three persons was detected during the day. On 28 June the movement of one and two persons was detected during the day (Figure 12), (Table 1). The drawback of this method of using the FBG sensor to detect the number of occupants in the monitored space is the lack of information on the occupancy of the monitored space of R104 in the SH. This drawback has been eliminated by adding CO_2_ sensors to rooms R104, R203 and R204 in experiment 2, which is described below.

### 3.2. Use of CO_2_ Sensors for Monitoring SH Space Occupancy

CO_2_ sensors (R104 (BT12.09), R203 (BT12.10), R204 (BT12.11)) can be used to detect occupancy of the monitored spaces (rooms R104, R203, R204) in the SH (Figure 5 and Figure 6); these sensors are used to control the quality of the indoor environment in these rooms using BACnet technology in the SH (Figure 7). To control HVAC in SH (Figure 7), other CO_2_ sensors which provide “measurement at the fresh outdoor air inlet into QPA 2062 SH (BT 12.01) were used; measurement at the recirculation air inlet from SH spaces into the QPM 2162 heat recovery unit (BT 12.02); measurement at the recirculation air inlet from SH spaces into the QPA 2062 heat recovery unit (BT 12.03); measurement in the QFA 2060 heat recovery unit (BT 12.04); measurement at the recirculation and fresh air outlet from the heat recovery unit into the QFM 2160 SH (BT 12.05); measurement at the exhaust air inlet into the QFM 2160 recuperation unit (BT 12.06); measurement at the exhaust air outlet from the QPA 2062 recuperation unit (BT 12.07); measurement at the recirculation air inlet from SH spaces into the QPM 2162 heat recovery unit (BT 12.08)”.

**Discussion—Experiment 3.2:**

**Figure 13:**
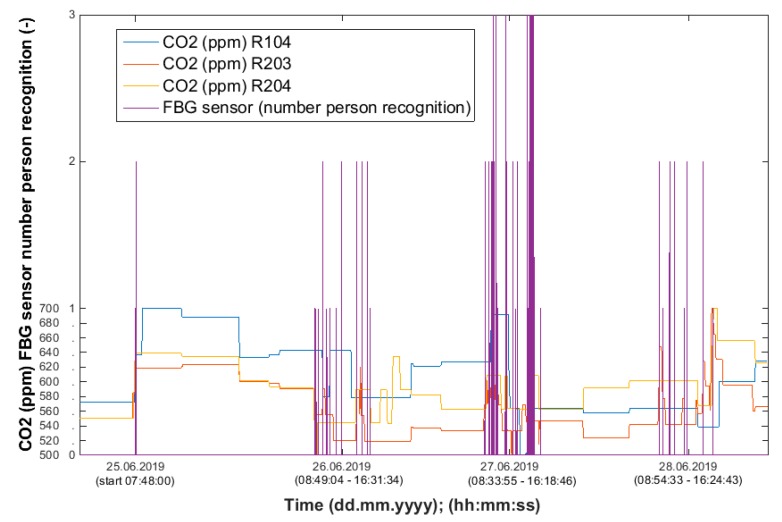
Waveforms of the CO_2_ concentration values measured in SH rooms R104 (BT 12.09), R203 (BT 12.10), R204 (BT 12.11) in order to detect the occupancy of the monitored spaces with the representation of the recognition of the number of occupants in room R104 using the FBG sensor.

**Figure 14:**
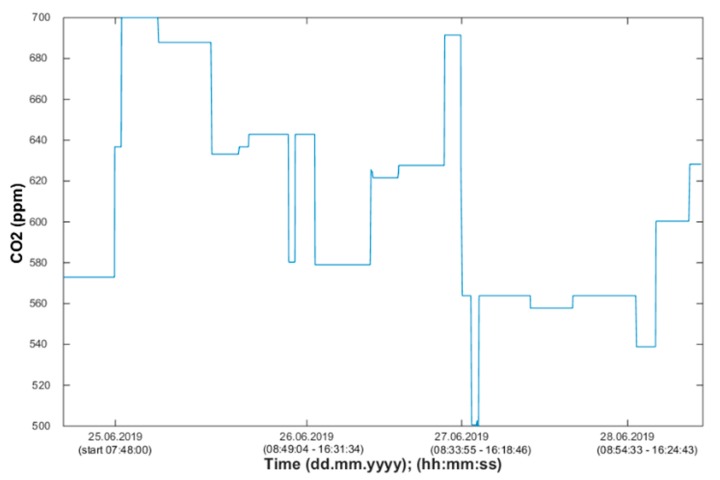
Waveforms of the CO_2_ concentration values measured in SH room R104 (BT 12.09).

**Figure 15:**
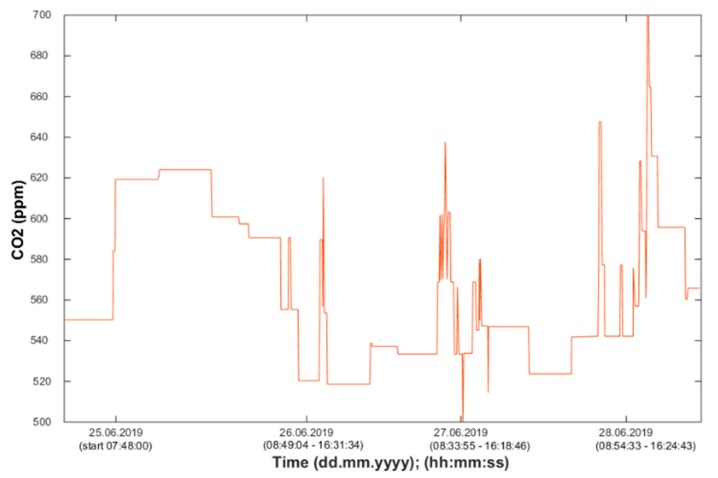
Waveforms of the CO_2_ concentration values measured in SH room R203 (BT 12.10).

**Figure 16:**
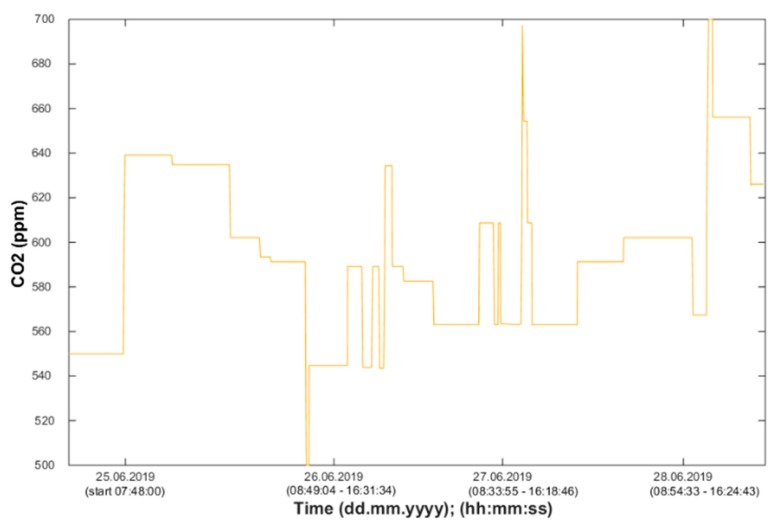
Waveforms of the CO_2_ concentration values measured in SH room R204 (BT 12.11).

Using the information from the FBG sensor (Figure 13), it is possible to unambiguously detect the number of occupants present in the monitored space. Based on the measured values (CO_2_ concentration), it is possible to detect the occupancy of the monitored SH spaces, the arrival of a person into the monitored room or the exit from the monitored space, or the length of stay in the monitored space (Figure 14, Figure 15 and Figure 16). The aforementioned procedure enables the unambiguous determination of the occupancy rate of the monitored SH spaces, indirectly by measuring common non-electrical quantities (CO_2_) within the operational–technical function control in the SH.

However, if the building is of an administrative type, the acquisition of CO_2_ sensors for dozens of rooms is a major investment. In market research, we ascertained that CO_2_ sensors are two to three times (in some cases, greater) more expensive than temperature and humidity sensors, which are, moreover, a common part of individual rooms in administrative buildings in the Czech Republic.

Due to the higher costs of acquiring CO_2_ sensors, we proposed the possibility of lowering the initial investment costs for larger administrative buildings by providing information about the occupancy of the individual rooms within the newly devised method of CO_2_ prediction. The mathematical method of ANN SCG was used to predict the waveform of CO_2_ concentration using the values measured from indoor temperature (*T*_in_) and indoor relative humidity (rH_in_) sensors. The experiments performed within the newly devised method are presented in the following text.

### 3.3. Experimental Verification of the Method with the Devised Artificial Neural Network (ANN) Scaled Conjugate Gradient (SCG)

**Figure 17:**
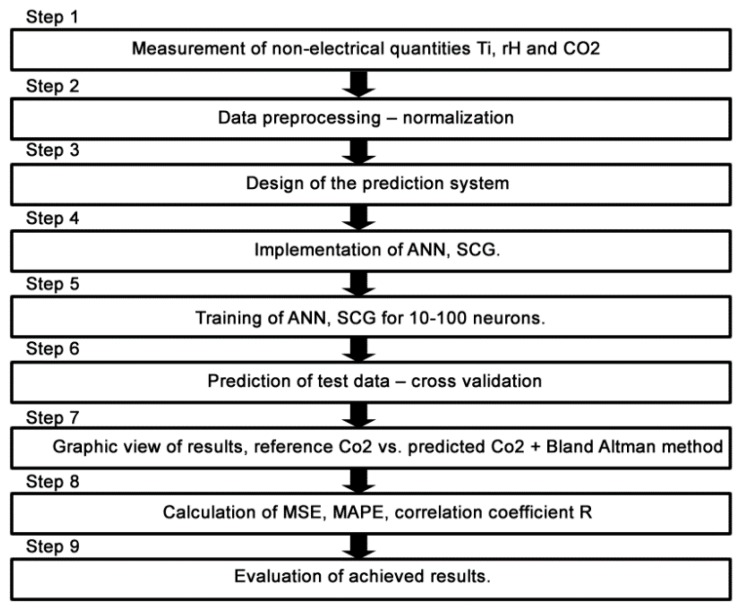
The waveform of the experiments performed on 26 June 2018, 27 June 2018, and 28 June 2018 for rooms R104, R203 and R204.

The conditions of experiments 3.3a and 3.3b were as follows. The experiments were performed for the waveforms of temperature (*T*_in_), relative humidity (rH_in_) and CO_2_ concentration measured on 26 June 2018, 27 June 2018, and 28 June 2018, (step 1) for rooms R104, R203 and R204 (Figure 17).

**Figure 18:**
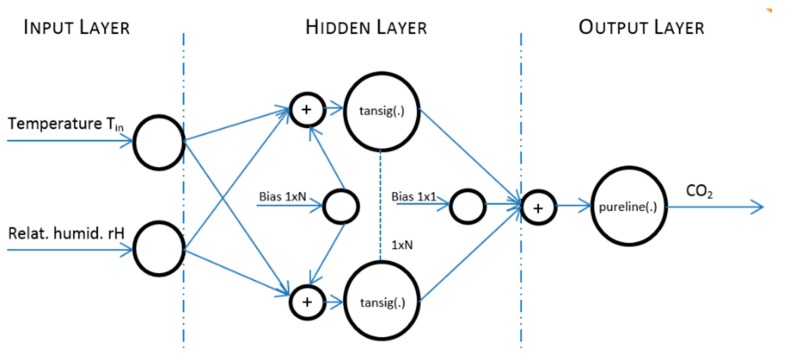
The architecture of the designed ANN SCG on test data measured in R203 from 25 June 2018 for two inputs *T*_in_ and rH_in_ without and FBG sensor for person presence measuring (PPM).

Furthermore, the measured data were pre-processed (step 2) (Figure 17). The design of the prediction system structure (step 3), the implementation (step 4) and ANN SCG training (step 5) for CO_2_ prediction using two input values of temperature (*T*_in_) and relative humidity (rH_in_) were carried out. The ANN SCG structure designed (Figure 18) for experiment 3.3a was trained (step 5) for the temperature (*T*_in_), relative humidity (rH_in_) and CO_2_ concentration values measured in room R203. The actual CO_2_ prediction (step 6) was implemented for the data measured in rooms R104, R203 and R204. To increase the accuracy of the newly devised method, an additional quantity from the FBG sensor containing the number of occupants in the monitored space of R104 was added to the original two input quantities. The ANN SCG structure designed for experiment 3.3b for CO_2_ prediction using three input quantities—temperature (*T*_in_), relative humidity (rH_in_) and the number of occupants (FBG sensor). The procedure for performing the experiments was the same as that described in Figure 17.

**Experiment 3.3a:**

**Figure 19:**
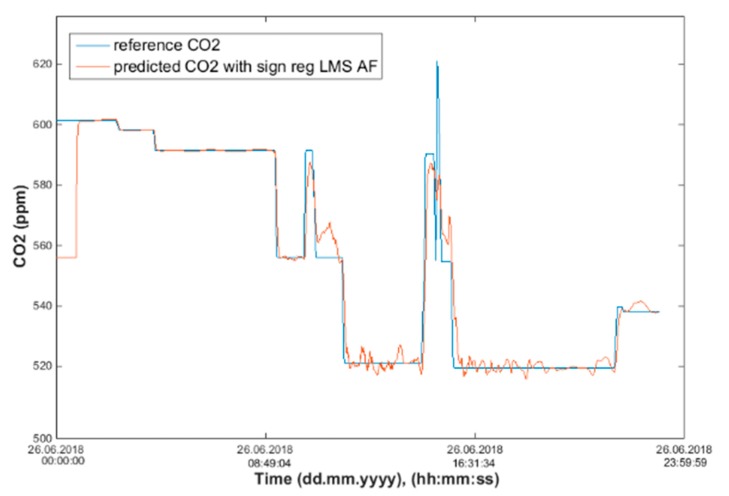
Comparison of the reference CO_2_ concentration waveform and predicted CO_2_ waveforms (SRLMS AF) from 26 June 2018 in R203 with an ANN with 100 neurons and SCG method trained with data from 25 June 2018 in R203.

**Figure 20:**
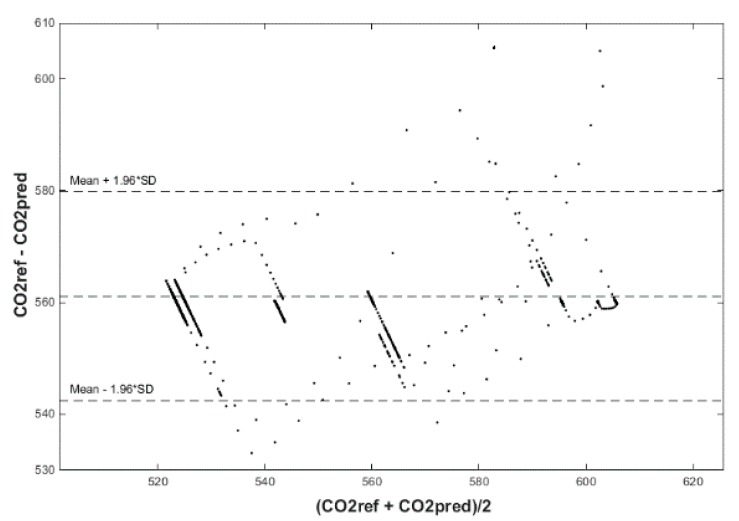
Bland–Altman plot for the reference and predicted CO_2_ waveforms (SRLMS AF) from 26 June 2018 in R203 with an ANN with 100 neurons and SCG method trained with data from 25 June 2018 in R203.

**Figure 21:**
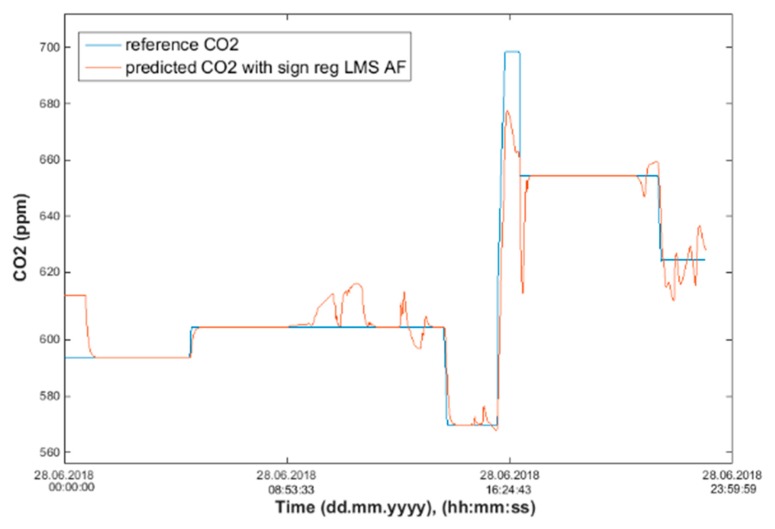
Comparison of the reference and predicted CO_2_ waveforms (SRLMS AF) from 28 June 2018 in R204 with an ANN with 40 neurons and SCG method trained with data from 25 June 2018 in R203.

**Figure 22:**
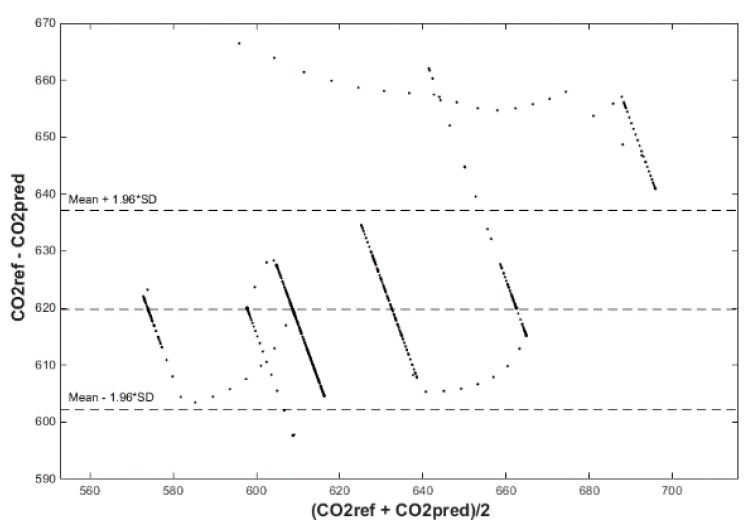
Bland–Altman plot for the reference and predicted CO_2_ waveforms (SRLMS AF) from 28 June 2018 in R204 with an ANN with 40 neurons and SCG method trained with data from 25 June 2018 in R203.

**Figure 23:**
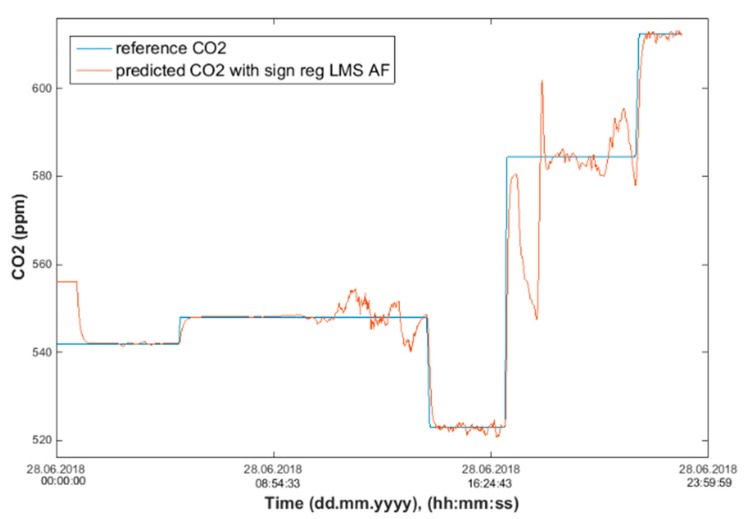
Comparison of the reference and predicted CO_2_ waveforms (SRLMS AF) from 28 June 2018 in R104 with an ANN with 60 neurons and SCG method trained with data from 25 June 2018 in R203.

**Figure 24:**
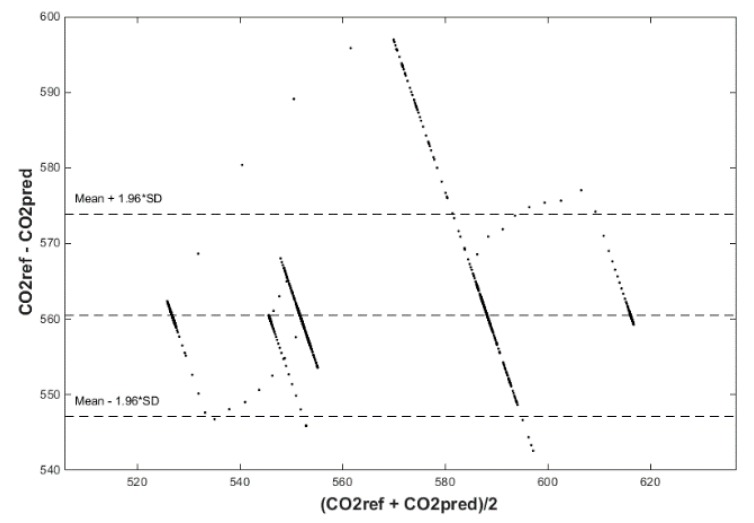
Bland–Altman plot for the reference and predicted CO_2_ waveforms (SRLMS AF) from 28 June 2018 in R104 with an ANN with 60 neurons and SCG method trained with data from 25 June 2018 in R203.

Input values, *T*_in_ and rH_in_, to ANN SCG for prediction of CO_2_ in rooms R203, R204, and R104 (Figure 18). Prediction of CO_2_ in rooms R203, R204, R104 for the dates 26, 27 and 28 June 2018 with inputs *T*_in_ and rH_in_, using learned ANN SCG (Figure 18) from 25 June 2018. Table 2, Table 3 and Table 4 show R, MSE and MAPE parameter values, followed by plots of reference and predicted CO_2_, as well as Bland–Altmann plots in rooms 203 (Figure 19 and Figure 20), 204 (Figure 21 and Figure 22) and 104 (Figure 23 and Figure 24).

**Experiment 3b:**

**Figure 25:**
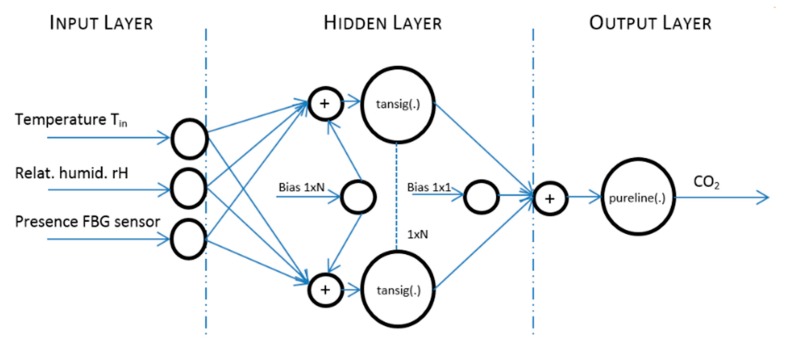
The architecture of designed ANN SCG on test data measured in R203 from 25 June 2018 for three inputs *T*_in_, rH_in_ and those of an FBG sensor for PPM.

**Figure 26:**
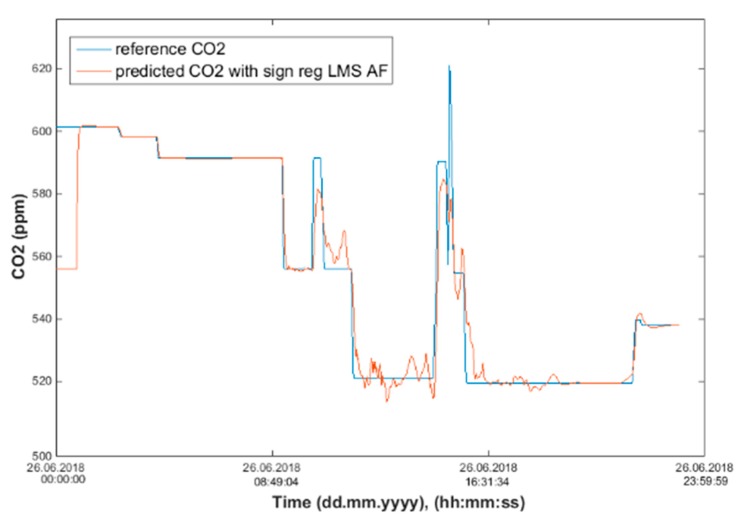
Comparison of the reference and predicted CO_2_ waveforms (SRLMS AF) from 26 June 2018 in R203 with an ANN with 60 neurons and SCG method trained with data from 25 June 2018 in R203.

**Figure 27:**
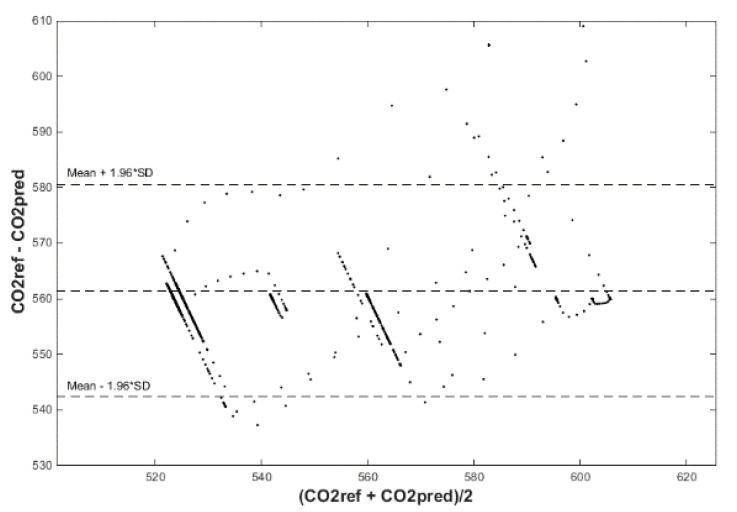
Bland–Altman for the reference and predicted CO_2_ waveforms (SRLMS AF) from 26 June 2018 in R203 with an ANN with 60 neurons and SCG method trained with data from 25 June 2018 in R203.

**Figure 28:**
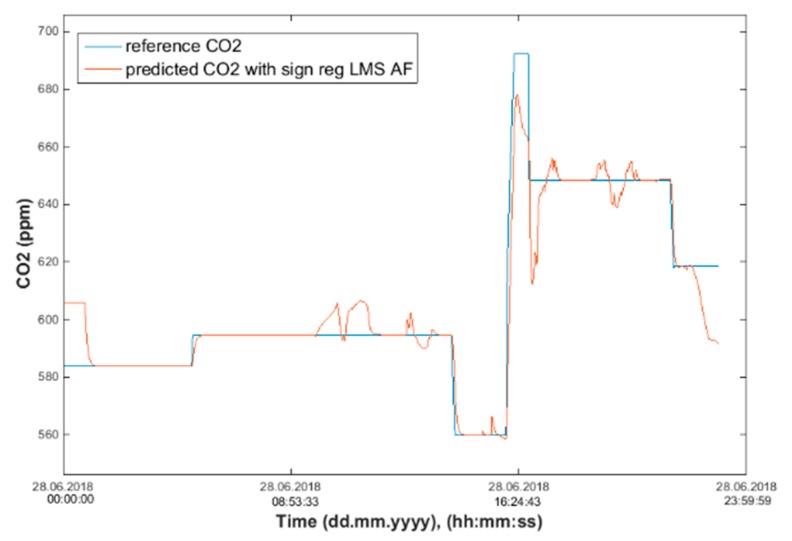
Comparison of the reference and predicted CO_2_ waveforms (SRLMS AF) from 28 June 2018 in R204 with an ANN with 90 neurons and SCG method trained with data from 25 June 2018 in R203.

**Figure 29:**
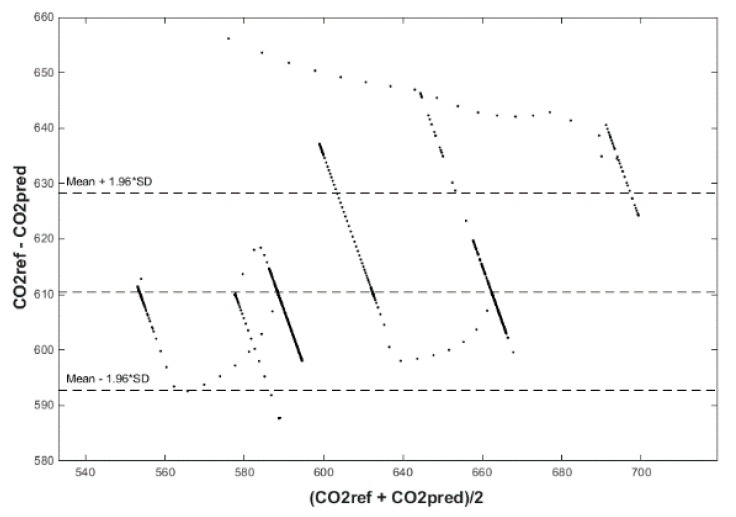
Bland–Altman for the reference and predicted CO_2_ waveforms (SRLMS AF) from 28 June 2018 in R204 with an ANN with 90 neurons and SCG method trained with data from 25 June 2018 in R203.

**Figure 30:**
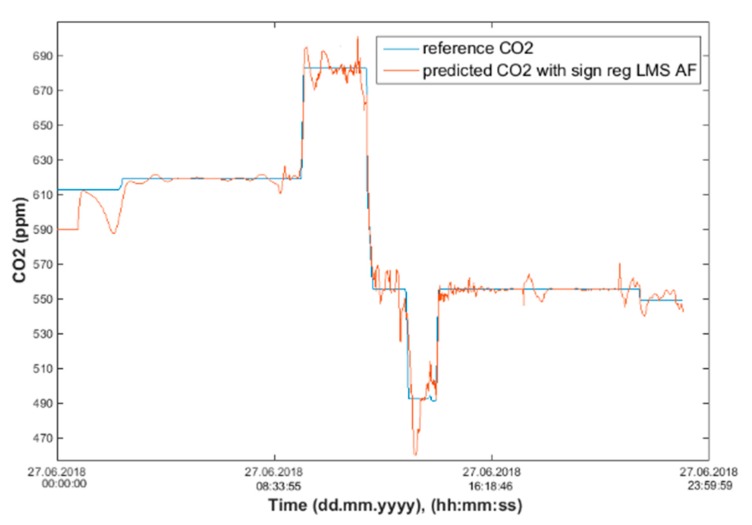
Comparison of the reference and predicted CO_2_ waveforms (SRLMS AF) from 27 June 2018 in R104 with an ANN with 60 neurons and SCG method trained with data from 25 June 2018 in R203.

**Figure 31:**
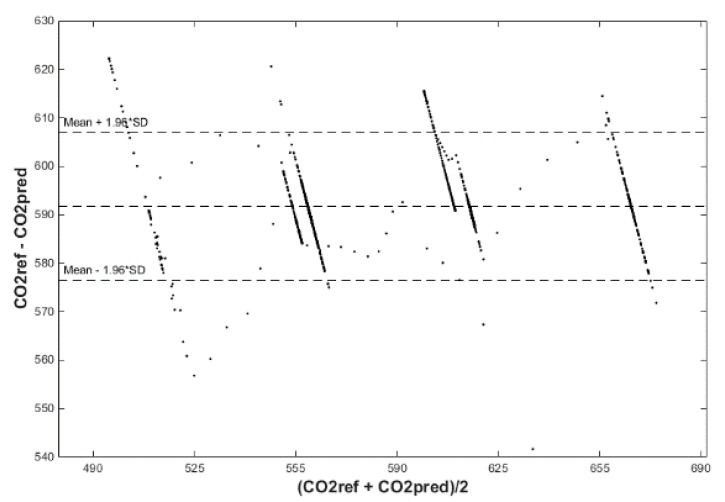
Bland–Altman for the reference and predicted CO_2_ waveforms (SRLMS AF) from 27 June 2018 in R104 with an ANN with 60 neurons and SCG method trained with data from 25 June 2018 in R203.

Our next experiment was performed with input values of *T*_in_ and rH_in_ and presence values from the FBG sensor input into ANN SCG for the prediction of CO_2_ in rooms R203, R204, R104 (Figure 25). Prediction of CO_2_ in rooms R203, R204, R104 for the dates 26, 27 and 28 June 2018 with input values from sensors *T*_in_, rH_in_ and FBG using learned ANN SCG (Figure 25) from 25 June 2018. Table 5, Table 6 and Table 7 show R, MSE and MAPE parameter values, followed by plots of reference and predicted CO_2_ as well as Bland–Altmann plots in rooms 203 (Figure 26 and Figure 27), 204 (Figure 28 and Figure 29) and 104 (Figure 30 and Figure 31). 

**Discussion—Experiments 3.3a and 3.3b:**

The best result within the designed three-day (26 June to 28 June 2018) experiments for the learned ANN SCG from 25 June 2018, in R203 (with *T*_in_ and rH_in_) and the prediction with cross-validation for 26 June 2018, in R203, R204, R104 (with *T*_in_ and rH_in_), 27 June 2018, in R203, R204, R104 (with *T*_in_ and rH_in_), 28 June 2018, in R203, R204, R104 (with *T*_in_ and rH_in_), without using the FBG sensor measurements, was detected for ANN SCG in room R203 (Table 2) with the following values: R = 91.6 (%), MSE = 0.47 × 10^−2^, MAPE = 25.59 × 10^−2^, for 100 ANN SCG neurons (Figure 19 and Figure 20). This is because ANN SCG learned for the data measured on 25 June 2018, in room R203 was used for CO_2_ prediction. By contrast, the worst calculated result was detected for ANN SCG in room R104 (Table 4), where R = 21.88 (%), MSE = 1.21 × 10^−2^ and MAPE = 21.47 × 10^−2^ for a 10 ANN SCG neurons. This is because room R104 was the furthest away from room R203 for which ANN SCG was learned.

As for the CO_2_ prediction, the best result within the designed three-day experiments using the FBG sensor for learned ANN SCG from 25 June 2018, in R203 (with *T*_in_, rH_in_ and the FBG sensor for PPM) and prediction with cross-validation for 26 June 2018, in R203, R204, R104 (with *T*_in_, rH_in_ and the FBG sensor for PPM), 27 June 2018, in R203, R204, R104 (with *T*_in_, rH_in_ and the FBG sensor for PPM), 28 June 2018, in R203, R204, R104 (with *T*_in_, rH_in_ and the FBG sensor for PPM) was detected for ANN SCG—number of neurons = 60 in room R203 (Figure 26 and Figure 27), (Table 5), where, on 26 June 2018, R = 92.81 (%), MSE = 0.40 × 10^−2^, and MAPE = 21.55 × 10^−2^. This is because ANN SCG learned for the data measured on 25 June 2018, in room R203 was used for CO_2_ prediction. By contrast, the worst calculated result was detected for ANN SCG in room R204 (Table 6), where R = 21.45 (%), MSE = 1.11 × 10^−2^, and MAPE = 20.39 × 10^−2^, for 10 ANN SCG neurons. This is because there was no FBG sensor in room R204 to detect the presence of persons.

**Figure 32:**
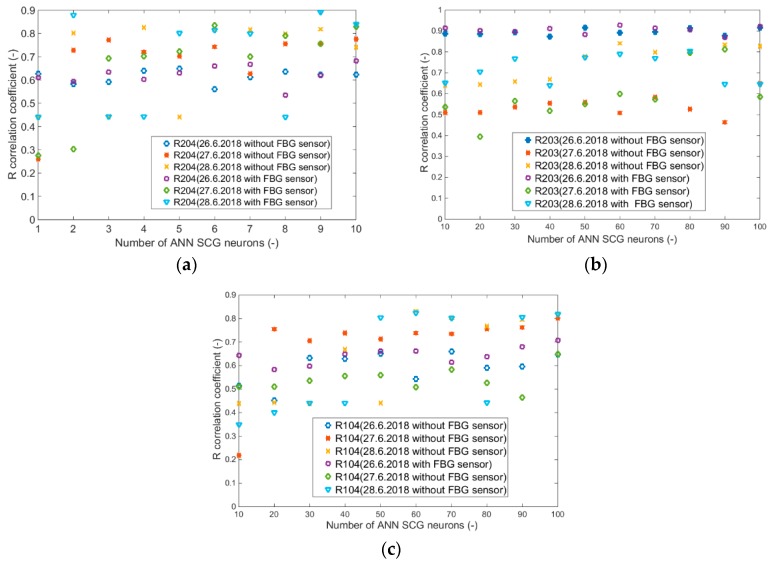
R-correlation coefficients for (**a**) room R203 with and without FBG, (**b**) room R204 with and without FBG, (**c**) room R104 with and without FBG.

**Figure 33:**
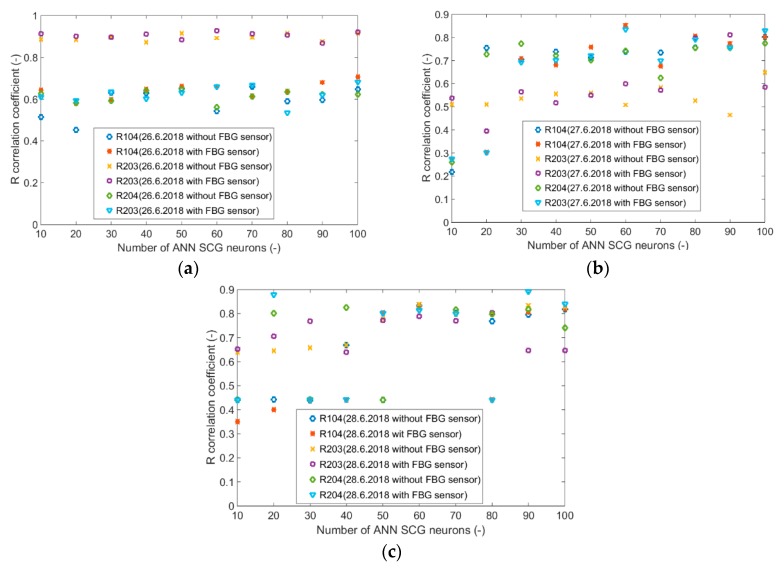
R-correlation coefficients for rooms R104, R203, R204 with and without FBG (**a**) 26 June 2018, (**b**) 27 June 2018, (**c**) 28 June 2018.

Based on the graphs illustrated in Figure 32a–c and Figure 33a–c, it can be stated that one universal learned ANN SCG cannot be used for the most accurate calculation of CO_2_ prediction for all SH spaces. In our experiments, ANN SCG was learned for the data measured in room R203 on 25 June 2018. The calculated values of the verification parameters measured (R coefficient, MSE and MAPE) in Table 2 and Table 5 (Figure 19, Figure 20, Figure 26 and Figure 27) indicate that, in order to achieve the greatest accuracy of CO_2_ prediction, it is necessary that ANN SCG is always learned for a specific room, for a specific monitored space in the SH. The expectations related to the use of the FBG sensor to increase the accuracy of CO_2_ prediction by means of ANN SCG were not fulfilled in the above-mentioned experiments (Figure 32 and Figure 33). However, the use of the FBG sensor in the SH is very useful from the point of view of a robust and stable solution for recognizing the number of occupants in the monitored SH spaces or intelligent administrative buildings. The mathematical method with ANN SCG is not the most suitable for the devised CO_2_ prediction method. For greater accuracy, it is necessary to implement filter algorithms [28] to remove additive noise from the predicted CO_2_ concentration waveform. Based on the results achieved and described above, further experiments on CO_2_ prediction will be conducted using more precise mathematical methods [29,30,31,32,33,34,37,38,39] within the IoT platform [40].

## 4. Conclusions

In order to detect the occupancy of the individual SH spaces by indirect methods (without using cameras), common operational and technical sensors were used in the experiments conducted to measure the indoor temperature, the indoor relative humidity and the indoor CO_2_ concentration within the BACnet technology for HVAC control in the SH.

Based on the experiments conducted in rooms R104, R203 and R204, it is possible to unambiguously detect the suitability of the sensors used for measuring the CO_2_ concentration in order to detect the occupancy and the length of stay in the monitored SH space. Moreover, the CO_2_ sensors used in the technology that were exclusively for HVAC control in the SH can be used to detect the occupancy and the length of stay in the monitored spaces, which will make the required information more accurate.

From the perspective of cost reduction, a method for predicting the CO_2_ concentration waveform by means of ANN SCG from the indoor temperature and indoor relative humidity values measured were devised and verified for the space location of an occupant (in rooms R104, R203, and R204) in the SH with the highest possible accuracy. The ANN SCG mathematical method used does not achieve such accuracy compared to the methods used in [28,29,30] and [34,37,38,39,40]. For selected experiments, nevertheless, the correlation coefficient in this article was greater than 90%. An increase in the correlation coefficient value can be achieved by using filter methods for suppressing additive noise from the predicted CO_2_ concentration waveform [28]. In the experiments conducted, we verified that, for each monitored space in the SH, the ANN SCG should be learned so that the CO_2_ prediction was as accurate as possible.

The FBG sensor was used to unambiguously detect the number of occupants in the monitored space of room R104. The experiments confirmed the suitability of using FBG sensors for reasons of robustness, accuracy and high reliability in the detection of the number of occupants moving in the monitored SH or Intelligent Building space without the need to deploy cameras. The experiments did not confirm the assumption that the input value of the presence of occupants added from the FBG sensor to the ANN SCG will make the prediction of the CO_2_ concentration using the ANN SCG method more accurate.

The application of FBG sensors in a passive SH is described. These sensors are electrically passive, while allowing the evaluation of multiple quantities at the same time, and with suitable encapsulation of FBG, the sensor is immune to EMI (electromagnetic interference). Due to its small size and weight, implementation in an SH is both straightforward and cost-effective. Using a spectral approach, tens of FBG sensors can be evaluated using a single evaluation unit [21].

In the next work, the authors will focus on verifying the practical implementation of the method devised for CO_2_ prediction in an SH for monitoring the occupancy of the SH within the IoT platform [40].

## Figures and Tables

**Table 1:** Unambiguous determination of the number of occupants on the staircase in room R104 in the SH.

Date	Time	Number of Occupants	Date	Time	Number of Occupants	Date	Time	Number of Occupants
(dd.mm.yyyy)	(hh:mm:ss)		(dd.mm.yyyy)	(hh:mm:ss)		(dd.mm.yyyy)	(hh:mm:ss)	
25 June 2018	7:48:07	1	27 June 2018	9:40:24	2	27 June 2018	14:52:53	2
25 June 2018	7:53:11	1	27 June 2018	9:44:09	3	27 June 2018	14:53:13	3
25 June 2018	7:53:13	2	27 June 2018	9:47:55	2	27 June 2018	14:56:13	2
25 June 2018	7:53:23	1	27 June 2018	9:51:23	1	27 June 2018	14:56:49	3
26 June 2018	8:49:04	1	27 June 2018	9:52:31	2	27 June 2018	15:01:32	2
26 June 2018	8:59:39	1	27 June 2018	10:04:16	3	27 June 2018	15:02:11	3
26 June 2018	9:21:15	1	27 June 2018	10:06:44	2	27 June 2018	15:12:12	2
26 June 2018	9:55:43	2	27 June 2018	10:08:47	1	27 June 2018	15:13:37	2
26 June 2018	10:29:44	1	27 June 2018	10:09:10	2	27 June 2018	15:13:41	3
26 June 2018	10:54:16	1	27 June 2018	10:21:31	1	27 June 2018	15:14:28	2
26 June 2018	11:49:18	1	27 June 2018	11:25:30	2	27 June 2018	15:14:50	3
26 June 2018	12:34:02	2	27 June 2018	11:29:22	3	27 June 2018	15:15:49	2
26 June 2018	12:35:40	1	27 June 2018	11:33:34	2	27 June 2018	15:22:19	3
26 June 2018	14:38:27	2	27 June 2018	11:36:08	1	27 June 2018	15:23:10	2
26 June 2018	15:24:31	1	27 June 2018	11:36:48	0	27 June 2018	16:18:22	1
26 June 2018	15:24:52	2	27 June 2018	12:28:48	1	27 June 2018	16:18:46	0
26 June 2018	16:07:57	1	27 June 2018	12:28:57	2	28 June 2018	8:54:33	1
26 June 2018	16:08:25	2	27 June 2018	12:53:14	1	28 June 2018	8:54:35	2
26 June 2018	16:31:34	1	27 June 2018	13:03:53	2	28 June 2018	9:22:11	1
27 June 2018	8:33:55	1	27 June 2018	14:25:57	1	28 June 2018	10:19:19	1
27 June 2018	8:35:22	2	27 June 2018	14:26:27	2	28 June 2018	10:20:05	2
27 June 2018	9:07:51	1	27 June 2018	14:29:00	3	28 June 2018	10:59:54	1
27 June 2018	9:09:36	2	27 June 2018	14:30:04	2	28 June 2018	11:00:07	2
27 June 2018	9:26:26	1	27 June 2018	14:33:03	1	28 June 2018	12:23:23	1
27 June 2018	9:30:32	2	27 June 2018	14:44:50	2	28 June 2018	12:45:23	2
27 June 2018	9:34:57	1	27 June 2018	14:45:09	1	28 June 2018	15:01:41	1
27 June 2018	9:35:50	2	27 June 2018	14:46:08	2	28 June 2018	15:01:57	2
27 June 2018	9:39:48	1	27 June 2018	14:51:29	3	28 June 2018	16:22:41	1

**Table 2:** Learned ANN SCG from 25 June 2018 in R203 (with *T*_in_ and rH_in_) and prediction with cross-validation for 26 June 2018 in R203 (with *T*_in_ and rH_in_), 27 June 2018 in R203 (with *T*_in_ and rH_in_), 28 June 2018 in R203 (with *T*_in_ and rH_in_).

	26 June 2018 in R203			27 June 2018 in R203			28 June 2018 in R203		
Number of Neurons ANN SCG	MSE	R	MAPE	MSE	R	MAPE	MSE	R	MAPE
	(-)	(-)	(-)	(-)	(-)	(-)	(-)	(-)	(-)
10	0.0062	0.8867	0.2858	0.006	0.5094	0.198	0.0206	0.6398	0.3275
20	0.0063	0.8846	0.289	0.006	0.5098	0.1992	0.0204	0.6449	0.3238
30	0.0058	0.8943	0.2872	0.0058	0.5355	0.1794	0.0198	0.6586	0.3265
40	0.0071	0.8728	0.3237	0.0056	0.5555	0.2036	0.0193	0.6687	0.3186
50	0.0047	0.9151	0.2171	0.0056	0.5591	0.1633	0.0138	0.7784	0.212
60	0.006	0.8918	0.3298	0.0061	0.5084	0.1906	0.0103	0.8401	0.1818
70	0.0059	0.8957	0.3025	0.0054	0.5824	0.1861	0.0127	0.7983	0.2091
80	0.0048	0.9145	0.2475	0.0059	0.5261	0.1845	0.0127	0.7988	0.2558
90	0.0069	0.877	0.1623	0.0065	0.4645	0.2097	0.0106	0.8346	0.2221
100	0.0047	0.916	0.2559	0.0047	0.6489	0.1614	0.0111	0.8266	0.1884

**Table 3:** Learned ANN SCG from 25 June 2018 in R203 (with *T*_in_ and rH_in_) and prediction with cross-validation for 26 June 2018 in R204 (with *T*_in_ and rH_in_), 27 June 2018 in R204 (with *T*_in_ and rH_in_), and 28 June 2018 in R204 (with *T*_in_ and rH_in_).

	26 June 2018 in R204			27 June 2018 in R204			28 June 2018 in R204		
Number of Neurons ANN SCG	MSE	R	MAPE	MSE	R	MAPE	MSE	R	MAPE
	(-)	(-)	(-)	(-)	(-)	(-)	(-)	(-)	(-)
10	0.0107	0.6276	0.2117	0.0112	0.2603	0.209	0.0187	0.4399	0.1699
20	0.0117	0.5816	0.2634	0.0057	0.7272	0.156	0.0087	0.8007	0.1154
30	0.0116	0.5914	0.2917	0.0049	0.7726	0.1232	0.0186	0.4429	0.1678
40	0.0104	0.6402	0.2231	0.0058	0.7213	0.1535	0.0074	0.825	0.1015
50	0.0103	0.6482	0.2425	0.0061	0.7017	0.1387	0.0186	0.4407	0.1692
60	0.0122	0.5606	0.2589	0.0054	0.7425	0.1463	0.0077	0.817	0.11
70	0.0111	0.6125	0.2503	0.0073	0.6261	0.1668	0.0077	0.8159	0.1065
80	0.0106	0.6354	0.2188	0.0052	0.7551	0.1133	0.0085	0.7971	0.1174
90	0.0111	0.6228	0.1671	0.0052	0.7555	0.1458	0.0077	0.8178	0.1078
100	0.0109	0.6232	0.2742	0.0048	0.7755	0.1404	0.0104	0.7416	0.1202

**Table 4:** Learned ANN SCG from 25 June 2018 in R203 (with *T*_in_ and rH_in_) and prediction with cross-validation for 26 June 2018 in R104 (with *T*_in_ and rH_in_), 27 June 2018 in R104 (with *T*_in_ and rH_in_), and 28 June 2018 in R104 (with *T*_in_ and rH_in_).

	26 June 2018 in R104			27 June 2018 in R104			28 June 2018 in R104		
Number of Neurons ANN SCG	MSE	R	MAPE	MSE	R	MAPE	MSE	R	MAPE
	(-)	(-)	(-)	(-)	(-)	(-)	(-)	(-)	(-)
10	0.0097	0.6714	0.3365	0.0121	0.2188	0.2147	0.0187	0.4399	0.1694
20	0.0102	0.6516	0.2259	0.0052	0.7541	0.1466	0.0186	0.4418	0.1713
30	0.0105	0.6398	0.2233	0.0061	0.7052	0.1395	0.0187	0.4396	0.1699
40	0.0099	0.6643	0.2074	0.0055	0.7389	0.144	0.0128	0.6684	0.1197
50	0.0098	0.6685	0.2099	0.0059	0.7136	0.1319	0.0186	0.4411	0.1692
60	0.0098	0.6697	0.2044	0.0055	0.7387	0.1544	0.0025	0.9135	0.0905
70	0.0099	0.6626	0.1937	0.0056	0.7345	0.1464	0.0082	0.8031	0.1089
80	0.0101	0.6542	0.2063	0.0051	0.7567	0.1134	0.0096	0.7676	0.1121
90	0.0095	0.6822	0.1859	0.0051	0.7613	0.1429	0.0085	0.7959	0.1216
100	0.01	0.6587	0.2005	0.0043	0.8027	0.1202	0.0077	0.8175	0.1041

**Table 5:** Learned ANN SCG from 25 June 2018 in R203 (with *T*_in,_ rH_in_ and an FBG sensor for PPM) and prediction with cross-validation for 26 June 2018 in R203 (with *T*_in,_ rH_in_ and an FBG sensor for PPM), 27 June 2018 in R203 (with *T*_in,_ rH_in_ and an FBG sensor for PPM), 28 June 2018 in R203 (with *T*_in,_ rH_in_ and an FBG sensor for PPM).

	26 June 2018 in R203			27 June 2018 in R203			28 June 2018 in R203		
Number of Neurons ANN SCG	MSE	R	MAPE	MSE	R	MAPE	MSE	R	MAPE
	(-)	(-)	(-)	(-)	(-)	(-)	(-)	(-)	(-)
10	0.0048	0.9139	0.2285	0.006	0.5094	0.198	0.0201	0.6521	0.2644
20	0.0055	0.9013	0.2814	0.006	0.5098	0.1992	0.0176	0.7052	0.2928
30	0.0057	0.8976	0.301	0.0058	0.5355	0.1794	0.0144	0.7684	0.2296
40	0.0049	0.9117	0.2189	0.0056	0.5555	0.2036	0.0206	0.64	0.3237
50	0.0064	0.8835	0.2802	0.0056	0.5591	0.1633	0.0141	0.7729	0.2079
60	0.004	0.9281	0.2255	0.0061	0.5084	0.1906	0.0132	0.7894	0.2
70	0.0048	0.9135	0.2377	0.0054	0.5824	0.1861	0.0142	0.7704	0.2353
80	0.0052	0.906	0.2541	0.0059	0.5261	0.1845	0.0124	0.8038	0.2117
90	0.0074	0.8684	0.2987	0.0065	0.4645	0.2097	0.0203	0.6467	0.3084
100	0.0044	0.9218	0.1693	0.0047	0.6489	0.1614	0.0203	0.6465	0.3002

**Table 6:** Learned ANN SCG from 25 June 2018 in R203 (with *T*_in,_ rH_in_ and an FBG sensor for PPM) and prediction with cross-validation for 26 June 2018 in R204 (with *T*_in,_ rH_in_ and an FBG sensor for PPM), 27 June 2018 in R204 (with *T*_in,_ rH_in_ and an FBG sensor for PPM), 28 June 2018 in R204 (with *T*_in,_ rH_in_ and an FBG sensor for PPM).

	26 June 2018 in R204			27 June 2018 in R204			28 June 2018 in R204		
Number of Neurons ANN SCG	MSE	R	MAPE	MSE	R	MAPE	MSE	R	MAPE
	(-)	(-)	(-)	(-)	(-)	(-)	(-)	(-)	(-)
10	0.0111	0.6096	0.0312	0.0111	0.2745	0.2039	0.0187	0.4407	0.172
20	0.0115	0.5939	0.2684	0.0111	0.302	0.2044	0.0053	0.8785	0.1011
30	0.0106	0.6343	0.2093	0.0063	0.6936	0.1521	0.0186	0.4421	0.1699
40	0.0113	0.6022	0.2116	0.0061	0.702	0.1549	0.0186	0.4422	0.1709
50	0.0107	0.6311	0.2047	0.0058	0.7221	0.1323	0.0082	0.8024	0.1139
60	0.01	0.6595	0.1621	0.0037	0.8352	0.1111	0.0078	0.8139	0.101
70	0.0098	0.6679	0.2093	0.0062	0.6994	0.1459	0.0084	0.7997	0.109
80	0.013	0.5346	0.2705	0.0045	0.7912	0.1289	0.0186	0.4409	0.1695
90	0.0109	0.6201	0.2235	0.0052	0.756	0.1452	0.0048	0.8912	0.0891
100	0.0095	0.6812	0.1807	0.0038	0.8289	0.1018	0.0068	0.8397	0.0948

**Table 7:** Learned ANN SCG from 25 June 2018 in R203 (with *T*_in,_ rH_in_ and an FBG sensor for PPM) and prediction with cross-validation for 26 June 2018 in R104 (with *T*_in,_ rH_in_ and an FBG sensor for PPM), 27 June 2018 in R104 (with *T*_in,_ rH_in_ and an FBG sensor for PPM), 28 June 2018 in R104 (with *T*_in,_ rH_in_ and an FBG sensor for PPM).

	26 June 2018 in R104			27 June 2018 in R104			28 June 2018 in R104		
Number of Neurons ANN SCG	MSE	R	MAPE	MSE	R	MAPE	MSE	R	MAPE
	(-)	(-)	(-)	(-)	(-)	(-)	(-)	(-)	(-)
10	0.0104	0.6432	0.2095	0.0112	0.2735	0.2028	0.0231	0.3505	0.2229
20	0.0117	0.5828	0.0638	0.0109	0.3042	0.2001	0.0195	0.4006	0.2088
30	0.0115	0.5987	0.2458	0.006	0.7079	0.151	0.0187	0.4416	0.1723
40	0.0103	0.6482	0.2155	0.0065	0.6818	0.1532	0.0187	0.4404	0.173
50	0.01	0.6612	0.2053	0.0051	0.7592	0.1147	0.0082	0.8034	0.1133
60	0.01	0.6613	0.2	0.0033	0.8534	0.0968	0.0074	0.8245	0.0953
70	0.0114	0.6135	0.2786	0.0066	0.6759	0.1525	0.0083	0.8015	0.108
80	0.0105	0.6379	0.2151	0.0042	0.8063	0.0555	0.0186	0.443	0.1678
90	0.0095	0.6799	0.1926	0.0048	0.7748	0.1293	0.0082	0.8052	0.1112
100	0.0089	0.7067	0.1906	0.0043	0.8026	0.103	0.0076	0.8194	0.1059

## Abbreviations

The following abbreviations are used in this manuscript:

(ANN)	artificial neural network
(FBG)	fiber Bragg grating
(SCG)	scaled conjugate gradient
(LMS)	least mean squares
(SRLMS)	signed—regressor least mean squares algorithm
(AF)	adaptive filter
(SH)	smart home
(SW)	software
(CO2)	carbon dioxide
(rH_in_)	relative humidity indoor
(*T*_in_)	temperature indoor
R104	room 104 in the smart home
R203	room 203 in the smart home
R204	room 204 in the smart home
(HVAC)	heating, ventilation and air conditioning
(IoT)	internet of things
(MSE)	mean squared error
(MAPE)	average absolute percentage error
(R)	correlation coefficient
(BACnet)	data communication protocol for building automation and control networks
(PPM)	person presence measuring
